# High-Efficiency Dynamic Terahertz Deflector Utilizing a Mechanically Tunable Metasurface

**DOI:** 10.34133/research.0274

**Published:** 2023-12-01

**Authors:** Zhenci Sun, Chao Liang, Chen Chen, Xiayu Wang, Enze Zhou, Xiaomeng Bian, Yuanmu Yang, Rui You, Xiaoguang Zhao, Jiahao Zhao, Zheng You

**Affiliations:** ^1^ Department of Precision Instrument, Tsinghua University, Beijing 100084, China.; ^2^ State Key Laboratory of Precision Measurement Technology and Instruments, Tsinghua University, Beijing 100084, China.; ^3^ Beijing Advanced Innovation Center for Integrated Circuits, Beijing 100084, China.; ^4^ School of Instrument Science and Opto-Electronic Engineering, Beijing Information Science and Technology University, Beijing 100016, China.

## Abstract

Terahertz (THz) wave manipulation, especially the beam deflection, plays an essential role in various applications, such as next-generation communication, space exploration, and high-resolution imaging. Current THz optical components and devices are hampered by their large bulk sizes and passive responses, limiting the development of high-performance, miniaturized THz microsystems. Tunable metasurfaces offer a powerful dynamic optical platform for controlling the propagation of electromagnetic waves. In this article, we presented a mechanically tunable metasurface (MTM), which can achieve terahertz beam deflection and vary the intensity of the anomalous reflected terahertz wave by changing the air gap between the metallic resonator (MR) array with phase discontinuities and Au ground plane. The absence of lossy spacer materials substantially enhances deflection efficiency. The device was fabricated by a combination of the surface and bulk-micromachining processes. The THz beam steering capability was characterized using terahertz time domain spectroscopy. When the air gap is 50 μm, the maximum deflection coefficient reaches 0.60 at 0.61 THz with a deflection angle of ~44.5°, consistent with theoretical predictions. We further established an electrically tunable miniaturized THz device for dynamic beam steering by introducing a micro voice coil motor to control the air gap continuously. It is shown that our designed MTM demonstrates a high modulation depth of deflection coefficient (~ 62.5%) in the target steered angle at the operating frequency. Our results showcase the potential of the proposed MTM as a platform for high-efficiency THz beam manipulation.

## Introduction

The terahertz frequency range (0.1 to 10 THz) has garnered significant attention due to the promising application in high-speed wireless communication [1–3], high-resolution imaging [4–6], space exploration [7,8], biological sensing [9–11], and security detection [12,13], among others. Therefore, dynamic terahertz beam manipulation technologies, including beam steering and beam modulation, have become crucial for advancing these applications [14–17]. Conventional terahertz beam steering techniques involve reconfiguring electromagnetic (EM) wave propagation through various mechanisms, such as mechanic scanning lenses [18], flip mirrors [19], or phased arrays [20]. However, these conventional devices suffer from drawbacks like large bulky sizes, high power consumption, and complex signal feeding systems, posing significant challenges for achieving terahertz wavefront modulation at a subwavelength scale and achieving miniaturized systems or microsystems [21,22].

Metasurfaces, consisting of subwavelength meta-atoms, offer extensive design flexibility for developing compact functional devices to manipulate the EM waves [23–25]. The introduction of generalized Snell’s law also provides a convenient and effective design methodology for ultrathin metasurfaces to control the propagation of EM waves, such as beam steering, focusing, and orbital angular momentum generation [26]. In the microwave range, active electronic devices (varactors, diodes, and semiconductor switches) were employed to change the phase response distribution of metasurface array, enabling the dynamic THz beam steering and focusing [27,28]. However, when the operating wavelength shifts to the THz regime, the stimulated response of the electron becomes relatively weak, and the high losses of active electronic devices may constrain the phase shift [29].

Tunable metasurfaces have been extensively studied in the terahertz band, and they can be categorized into 2 main approaches: (a) tuning the properties of constituent materials by external excitations [30–32], and (b) changing the dimension parameters and geometric structures of meta-atoms by mechanical deformation [33–35]. In comparison to terahertz tunable metasurfaces enabled by modifying the constituent material properties, mechanically dynamic metasurfaces have shown great development prospects due to the high-power handling capability [36]. The mechanical approach can manipulate the near-field interactions between meta-atoms significantly to achieve large tunability, especially the high modulation depth and broad frequency tuning range [37]. Dynamic metasurfaces based on micro-electro-mechanical system (MEMS) have shown great development prospects for realizing efficient, broadband, and fast wavefront shaping in the optical regime [38,39]. Besides, the absence of constituent materials will be conducive to reduce dissipated energy and intrinsic losses inside the meta-atom, improving the efficiency of terahertz beam manipulation. Therefore, dynamic metasurfaces based on the mechanically tunable approach provide a flexible and efficient platform for manipulating THz waves with minimized parasitic effects [40].

In this study, we proposed a terahertz beam deflector based on the mechanically tunable metasurface (MTM) composed of 2 silicon chips spaced by an air gap. The MTM is designed to manipulate terahertz wavefronts and enable continuous modulation of deflection amplitude. Using the equivalent circuit model (ECM), we theoretically demonstrated that meta-atoms within the MTM exhibit reflection coefficient exceeding 0.95, covering a phase span of 360° when a substantial air gap (≥50 μm) is maintained. A subarray consisting of 6 air-spaced meta-atoms with a uniform phase gradient is selected for precise THz beam steering. The device was fabricated by 2 wafer processes, and characterized using an angle-resolved terahertz time domain spectroscopy (THz-TDS) system. Our device achieves a maximum deflection coefficient of 0.60 at the operating frequency of 0.61 THz with an output angle of approximately 44.5°. In addition, we demonstrate anomalous reflection coefficient modulation depth of 62.5% at 44° by continuously adjusting the air gap through applied currents ranging from 40 to 80 mA. This work presents a practical and effective solution for active terahertz beam steering through the utilization of a metasurface-based microsystem featuring a tunable air gap. We believe that the concept of MTM offers a general solution for reconfigurable intelligent surfaces (RISs) at terahertz and higher frequencies, which potentially have vast applications in high-speed communication and other information technologies.

## Results

### Design

**Fig. 1.**
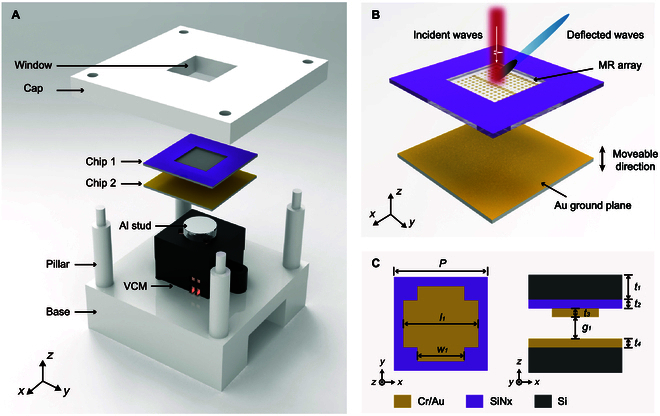
Introduction of the mechanically tunable metasurface (MTM). (A) Exploded view of the developed MTM. (B) Illustration of the beam steering by the tunable air gap. The blue and black THz beams represent deflected waves with high and low intensities, respectively. The HR silicon layer in Chip 1 is set as a transparent material to facilitate the view of the metallic resonator array below. (C) Schematic diagram of air-spaced meta-atom in the planar (*x*–*y*) and cross (*x*–*z*) section.

The proposed tunable terahertz beam deflector is composed of a metallic resonator array chip (Chip 1), a gold-coated silicon substrate (Chip 2), a micro voice coil motor (VCM), and a 3-dimensional (3D) printed skeleton structure, as shown in Fig. 1A. Chip 1 is attached to the backside of the top cap, while Chip 2 is connected to the VCM by a solid stud. As shown in Fig. 1B, the metallic resonator array is located on the back of Chip 1, and Chip 2 can move upward and downward in the *Z* direction by applying currents to the VCM. The air gap between the metallic resonator array and Au ground plane can be varied and continuously driven by electrical stimulation, forming an MTM. The terahertz beam is normally incident onto the MTM through a window. When the air gap is equal to the optimal design value, the phase gradient of meta-atom is uniform, and the phase response can cover 360°. According to the generalized Snell’s law, the THz beam will deflect to a designed angle (α), as shown in Fig. S1A. Owing to the removal of the lossy spacer materials, the deflection efficiency of air-spaced metasurface can be improved significantly. When the air gap is close to zero, the phase responses of all meta-atoms are approximately equal, and phase discontinuities in the metasurface vanish. In such condition, the THz beam will reflect vertically, and the intensity of the deflected beam in the angle (α) will drop particularly low, as shown in Fig. S1B. Therefore, we can vary the amplitude of the deflected THz beam by changing the air gap between the metallic resonator array and Au ground plane. As shown in Fig. 1C, the air-spaced meta-atom consists of a cross-shaped metallic resonator patterned on the backside of a high-resistivity (HR) silicon, which is covered by a silicon nitride membrane.

To investigate the resonance nature of the metasurfaces, we calculated the surface current and electric field distributions of meta-atom at the corresponding resonance frequencies by numerical simulation. A unit cell boundary condition and normal incidence were applied to the simulation model. The permittivity and conductivity of the HR silicon were set as 11.9 and 0.005 S/m, respectively. The permittivity of the silicon nitride was 7.6 + i0.04 [41]. Gold was modeled as lossy metal with a conductivity of 4.56×10^7^ S/m, and the interlayer was considered as vacuum. The periodicity of meta-atom (*P*) was set as 120 μm, and the air gap (*g*_1_) between the metallic resonator and Au ground plane was 50 μm. The length (*l*_1_) and width (*w*_1_) of the metallic resonator were 100 μm and 90 μm, respectively. The thickness values of each structural layer are listed in Table S1.

**Fig. 2.**
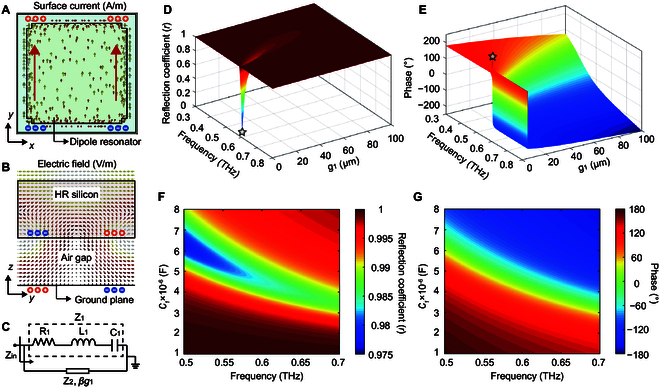
Equivalent circuit model of the air-spaced meta-atom. (A) The simulated surface current in the plane of metallic resonator (*x*–*y* plane). (B) Simulated electric field distribution of meta-atom in the cross-section (*y*–*z* plane). (C) The presented equivalent circuit model (ECM). (D) Calculated reflection coefficient and (E) phase responses versus air gaps (*g*_1_) and frequency. The star in panel D represents the point of perfectly impedance matching (lowest amplitude of reflection coefficient), while it represents the transition point from the overdamped region to the underdamped region in panel E. (F) Calculated reflection coefficient and (G) phase responses versus capacitances (*C*_1_) and frequency.

As shown in Fig. 2A, surface currents can be excited in the metallic resonator by the electric field component at the resonant frequency, and red arrows indicate the flow direction for y-polarized THz pulses. Surface currents along the metallic resonator are equivalent to a resistor and an inductor. The electric field is concentrated in the gap between 2 adjacent metallic resonators, indicating the contribution to the capacitance in the serially connected resistor-inductor-capactor (RLC) circuit. The electric field distribution of the air-spaced meta-atom in the *y*–*z* cross section is shown in Fig. 2B. The arrows (the direction of field lines) illustrate that meta-atom supports electric dipole moments with a separation of *g*_1_. Thus, an electric dipolar resonance mode appears due to the interlayer coupling between the metallic resonator and Au ground plane.

An ECM of meta-atom was further investigated to obtain a comprehensive understanding of the amplitude and phase responses, as shown in Fig. 2C. The top metallic resonator can be simplified as a circuit with a serially connected resistor (*R*_1_), inductor (*L*_1_), and capacitor (*C*_1_). A paralleled impedance (*Z_2_*) represents the interlayer coupling induced by the air gap. The reflection coefficient (*r*) of the meta-atom can be calculated from the impedance mismatch [42] (see Note S3 for derivation):

$$\begin{array}{c} r=\\ \frac{\omega^{2}L_{1}C_{1}Z_{0}+\omega C_{1}\sqrt{\mu_{0}/\varepsilon_{0}}\text{tan}\left(\frac{\omega \times g_{1}}{c}\right)\left(Z_{0}-R_{1}\right)-Z_{0}-i\left[\omega^{2}L_{1}C_{1}\sqrt{\mu_{0}/\varepsilon_{0}}\text{tan}\left(\frac{\omega \times g_{1}}{c}\right)+\omega R_{1}C_{1}Z_{0}-\sqrt{\mu_{0}/\varepsilon_{0}}\text{tan}\left(\frac{\omega \times g_{1}}{c}\right)\right]}{-\omega^{2}L_{1}C_{1}Z_{0}-\omega C_{1}\sqrt{\mu_{0}/\varepsilon_{0}}\text{tan}\left(\frac{\omega \times g_{1}}{c}\right)\left(Z_{0}+R_{1}\right)+Z_{0}-i\left[\omega^{2}L_{1}C_{1}\sqrt{\mu_{0}/\varepsilon_{0}}\text{tan}\left(\frac{\omega \times g_{1}}{c}\right)-\omega R_{1}C_{1}Z_{0}-\sqrt{\mu_{0}/\varepsilon_{0}}\text{tan}\left(\frac{\omega \times g_{1}}{c}\right)\right]} \end{array}$$ (1)

where *ω* is the operation frequency in radians, *Z*_0_ is the wave impedance of vacuum, *c* is the velocity of light, *g*_1_ is the air gap between the metallic resonator and the Au ground plane, and *μ*_0_ and *ε*_0_ are the permeability and permittivity of the vacuum, respectively. For our designed meta-atom with a 50-μm air gap, calculated results were resistance *R*_1_ (1.09 Ω), self-inductance *L*_1_ (0.11 nH), and capacitance *C_1_* (0.69 fF) retrieved by Eq. 1.

To investigate the mechanism of the tunable response, we varied only one of the electrical parameters in Eq. 1 without changing other values to study its influence on the EM responses. The paralleled impedance (*Z*_2_) can be directly calculated by plugging the air gap (*g*_1_) into Eq. S2. As the air gap increases from 0 to 100 μm, *Z*_2_ increases and the resonant frequency of the meta-atom blueshifts as shown in Fig. 2D. The reflection coefficient at the resonance frequency, or namely the *r* dip, reaches a minimum at a critical separation (~4.6 μm) indicating a perfect impedance match (shown as the star), and then increases above 0.9 when the air gap is larger than 30 μm. Figure 2E illustrates that the phase response transits from the overdamped region to the underdamped region at this specific air gap (represented by the star). The phase span of the meta-atom can undergo a 360° coverage in the underdamped state, while it is less than 180° range in the overdamped state. As shown in Fig. 2F, the increase in capacitance *C*_1_ (0.1 to 0.8 pF) contributes to a decrease in resonance frequency, and the reflection coefficient is consistently larger than 0.97. It can be observed that when the air gap is large enough, the phase span can cover a 360° range across the resonant frequency without reducing the reflection coefficient. Therefore, the theoretical analysis based on ECM is instructive for designing high-efficiency THz beam deflection metasurface.

**Fig. 3.**
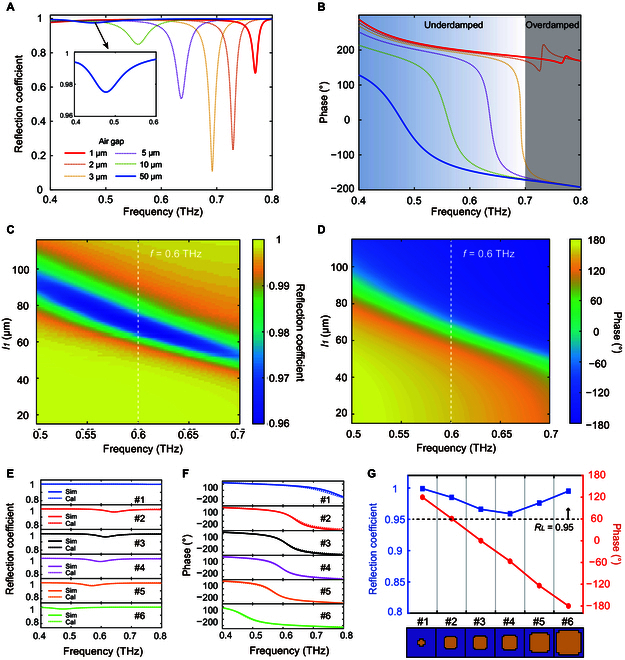
Simulated meta-atom properties. (A) The simulated reflection coefficient (*r*) and (B) phase responses of the air-spaced meta-atom for 6 different air gaps (*g*_1_ = 1, 2, 3, 5, 10, and 50 μm). (C) The simulated reflection coefficient and (D) phase responses of the air-spaced meta-atom versus metallic resonator length (*l*_1_) and frequency. (E) The simulated reflection coefficient and (F) phase responses of the selected 6 meta-atoms ranging from 0.4 to 0.8 THz. (G) The reflection coefficient (blue dot line) and the phase shift (red dot line) at the operating frequency (0.6 THz) of the selected 6 meta-atoms in a subarray. The reflection coefficient of all 6 air-spaced meta-atoms is greater than 0.95.

Numerical simulations were then conducted to investigate the effects of structural parameters (*g*_1_, *l*_1_) on the reflection coefficient and phase of the meta-atom. We changed only one of them while keeping other parameters as constant values. The initial geometrical sizes of the meta-atom were the same as previously (*P* = 120 μm, *t*_1_ = 60 μm, *t*_2_ = 0.2 μm, and *t*_3_ = *t*_4_ = 0.15 μm). As shown in Fig. 3A, increasing the air gap (*g*_1_) will lead to continuous blueshifts of the resonant frequency due to the increase in paralleled impedance *Z*_2_, which aligns with the calculated responses of meta-atoms based on the ECM shown in Fig. 2D. At a very small separation (<5 μm), the *r* dip reaches a minimum of approximately 0.02, and the phase spectrum undergoes an abrupt transition from the overdamped state to underdamped state as shown in Fig. 3B. When the air gap (*g*_1_) exceeds 10 μm, the reflection coefficient will be higher than 0.8, and the phase span can still cover a range of 360°. To achieve high deflection efficiency, a large air gap of 50 μm can be chosen.

The simulated coefficient and phase of the reflection response for varied *l*_1_ are shown in the colormaps in Fig. 3C and D, respectively. The metallic resonator length (*l*_1_) ranges from 15 μm to 115 μm with a step of 0.5 μm, and the metallic resonator width is defined as *w*_1_ = *l*_1_ − 10 μm. As the length (*l*_1_) increases, the dipolar resonance frequency decreases due to the increase of *C*_1_, and the *r* dip is consistently greater than 0.95 as shown in Fig. 3C. Figure 3D illustrates that the meta-atom with a 50*-*μm air gap operates in the underdamped region, and the phase span at resonance can cover a constant 360° range from 0.5 to 0.7 THz. Thus, the proposed ECM (Fig. 2F and G) can be employed to explain the influence of length (*l*_1_) on the EM responses of meta-atom (*g*_1_ = 50 μm). This is very advantageous for the design and optimization of highly efficient terahertz beam deflection devices.

Next, we selected 6 air-spaced meta-atoms (*g*_1_ = 50 μm) with a high reflection coefficient (>0.95) to form a subarray with a linear phase gradient of 60° at 0.6 THz, as shown in Fig. 3E to G. Detailed geometric parameters of these meta-atoms are listed in Table. Quantitative fitting parameters, including *R*_1_, *L*_1_, and *C*_1_, in the ECM are described in Table S2. The agreement between the calculation (dashed lines) and simulation (solid lines) validates the ECM, as illustrated in Fig. 3E and F. According to the generalized Snell’s law, the deflection angle (*θ_d_*) of an air-spaced metasurface can be expressed by

$$\left|\text{sin}\theta_{d}-\text{sin}\theta_{0}\right|=\frac{\lambda_{0}}{n\times P}$$ (2)

**Table.** Geometric parameters of meta-atoms

	#1	#2	#3	#4	#5	#6
*l*_1_ (μm)	19	60	65.5	69	75	101
*w*_1_ (μm)	9	50	55.5	59	65	91

where *θ*_0_ is the specular reflection angle, *λ*_0_ is the operating wavelength, *n* is the number of meta-atoms in a subarray, and *P* is the periodicity of the meta-atom. For our designed devices, *λ*_0_ = 500 μm, *n* = 6, and *P* = 120 μm. Based on Eq. 2, the deflection angle of the THz beam under normal incidence was calculated as approximately 44° at 0.6 THz.

**Fig. 4.**
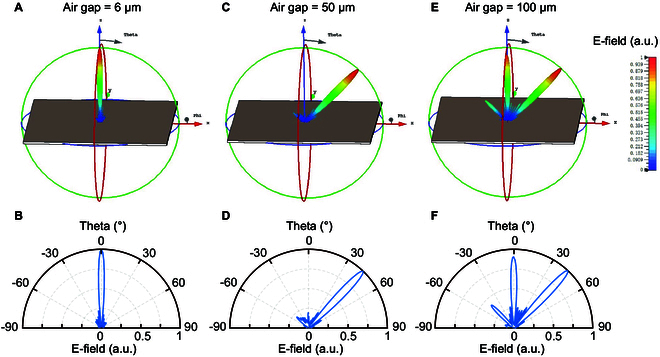
Simulated far-field scattering patterns of the metasurface array with different air gaps. 3D patterns: (A) 6 μm, (C) 50 μm, and (E) 100 μm. 1D polar charts: (B) 6 μm, (D) 50 μm, and (F) 100 μm.

According to the actual area size of devices (7.2×7.2 mm^2^), a full-wave simulation model including 60×60 meta-atoms has been established to analyze the terahertz energy distribution. A plane wave with linear polarization was set as source excitation to incident into the metasurface array normally. Figure 4 demonstrates the simulation results of the far-field scattering patterns of the air-spaced metasurface array with 3 different air gaps (6 μm, 50 μm, and 100 μm). For the metasurface-based deflector with a 6-μm air gap, the strong normal reflection appears in the far-field scattering pattern as shown in Fig. 4A and B. The anomalous reflection beam does not exist mainly because all the meta-atoms in the subarray have almost equal phase responses, and a 360° phase coverage is not achieved at 0.6 THz, as depicted in Fig. S2A. As the air gap increases to 50 μm, an oblique reflection beam can be observed at about +44° relative to the normal incidence, and the 3-dB beamwidth is around 8° as shown in Fig. 4C and D. It is noticed that the entire electric field is almost concentrated in this reflection angle, and the normalized magnitude of a normal reflection beam is less than 0.2. For the 100-μm air gap metasurface array, 3 reflection beams can be observed simultaneously at +44°, 0°, and −44° as shown in Fig. 4E and F. Due to the non-uniform phase gradient between the meta-atoms (Fig. S2B), the electric field is not perfectly restricted in the target reflection angle, and the THz beam leakage occurs. Thus, the magnitude of the electric field for the anomalous reflection beam at +44° decreases as the air gap changes from 50 μm to 100 μm. Moreover, the effects of air gap on the terahertz beam at other diffraction orders (m = 0 and −1) have also been calculated through the full-wave simulation (see Note S6). As *g*_1_ increases from 6 to 200 μm, the increase of the 0 order diffraction coefficient is accompanied by the decrease of the +1 order diffraction coefficient, while the −1 order diffraction coefficient remains less than 0.2. By changing the air gap, we can achieve the effective beam steering and adjust the intensity of the deflection beam continuously at the target angle.

**Fig. 5.**
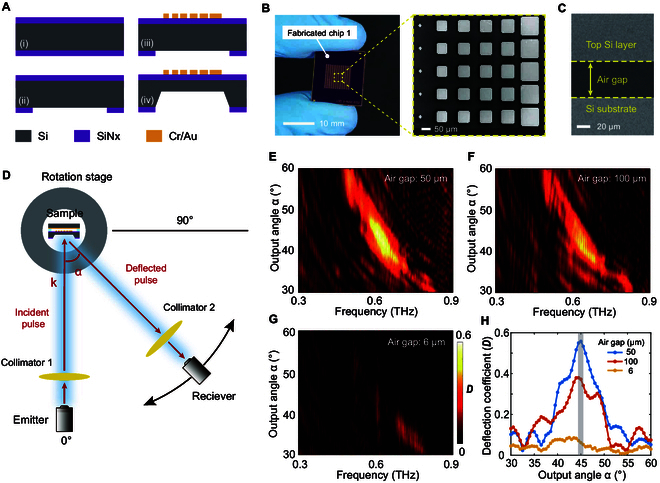
Fabrication and characterization of the metasurface. (A) Fabrication process of the Chip 1 including (i) SiNx LPCVD, (ii) ICP etching, (iii) Au patterning, and (iv) KOH wet etching. (B) Photograph of the fabricated Chip 1 and SEM image of the metallic resonator array. (C) The SEM cross-sectional image of the 50-μm air gap between the 2 chips. (D) The schematic diagram of the measurement THz-TDS system. Fourier transform of the received time-domain signals for 3 different devices with (E) 50 μm, (F) 100 μm, and (G) 6 μm air gap. (H) The deflection coefficient (*D*) versus output angle α at 0.6 THz for 3 different air gaps (*g*_1_ = 50, 100, and 6 μm).

The metasurface was fabricated using surface and bulk micro-machining processes, and Fig. 5A illustrates the fabrication process flow of Chip 1. The first wafer was a double-sided polished silicon wafer with HR (>10^4^ Ω·cm). It was coated by 200-nm-thick silicon nitride (SiNx) membrane on both sides by low-pressure chemical vapor deposition (LPCVD). The bottom SiNx layer was etched by inductively coupled plasma (ICP) etching to open windows for the potassium hydroxide (KOH) wet etching. Next, photolithography was performed on the top SiNx layer, and an array of metallic resonators were patterned using e-beam evaporation and lift-off processes. The first silicon wafer was then etched through using KOH anisotropic wet etching from the backside. Afterward, we can easily dice the first silicon wafer into small chips along the etched traces on the edge, as shown in Fig. S4. The surface morphology of the first silicon wafer after wet etching was measured by a 3D optical profilometer, and the thickness of the structural layer under the metallic resonator array was approximately 60 μm, as depicted in Fig. S5. Figure 5B illustrates that the metallic resonator array is constituted by 72 × 72 meta-atoms; i.e., there are 12 × 72 subarrays.

The 150-nm-thick gold ground plane was deposited on the second silicon wafer (500 μm thick) through e-beam evaporation. Then, the second silicon wafer was sliced into square pieces as Chip 2, which has the same area as Chip 1. To demonstrate the effect of the air gap on the beam steering, we bonded 2 silicon chips and fabricated several samples with 3 different air gaps (6 μm, 50 μm, and 100 μm) (see Note S8 for the specific description of the process flow). The SEM images of the cross-section of the fabricated devices are shown in Fig. 5C and Fig. S7. Figure 5C illustrates that the air gap between the 2 silicon chips was measured as approximately 50 μm.

We employed the optical fiber-based THz-TDS system to characterize the far-field angular distribution of air-spaced metasurfaces, as illustrated in Fig. 5D. The THz emitter and receiver modules were mounted at the ends of two arms in the rotation stage. The fabricated device was fixed at the center of the rotation stage. The THz broadband pulse radiated by the fixed emitter is incident perpendicular to the metasurface through a collimator lens, while the anomalous reflection wave is collected by the rotated THz receiver. Note that the deflection angle (α) cannot be less than 20° to prevent the THz emitter and receiver from colliding.

The deflected wave was detected at an angle resolution of 0.5° by rotating the THz receiver in the range of 30° to 60°. Next, fast Fourier transform was applied to the measured time-domain signals to obtain the frequency spectra. The calculated frequency-domain signals were normalized with the reference signal from the transmission spectrum of air (see Note S10 for the specific measurement and calculation of reflection coefficient). Figure 5E to G show the far-field reflection frequency spectra over the frequency range of 0.3 to 0.9 THz for the 3 samples (*g*_1_ = 50 μm, 100 μm, and 6 μm) at various output angles. As shown in Fig. 5E, when the air gap is 50 μm, the maximum deflection coefficient can reach 0.60 at 0.61 THz, and the deflection angle is around 44.5°, which agree well with the predicted results calculated by the generalized Snell’s law. As can be seen, the difference between the measured and calculated results is within 1°, indicating that our fabricated metasurface can achieve beam deflection as we design. Compared with the designed operation frequency of 0.6 THz, there is a tiny frequency shift of about 7 GHz, which can be attributed to the fabrication errors of the metallic resonator array and the non-uniform air gap. With the change of the air gap, the deflection coefficient distinctly reduces as shown in Fig. 5F and G. The experimental deflection spectra of the devices with a 50-μm and a 100-μm air gap exhibit a similar characteristic, and the deflected angle of the anomalous reflection beam remains almost unchanged, showing a good agreement with the simulation results.

At the operating frequency of 0.6 THz, the deflection coefficients of the devices with 3 different air gaps with respect to the scan angles (30° to 60°) are plotted in Fig. 5H. When the air gap is 50 μm, a deflection coefficient peak is around 0.56 at a deflected angle of 45°, and the 3-dB beamwidth is approximately 5°, indicating that it has good beam directivity. Besides, the anomalous refection coefficient of the device with a 100-μm air gap was measured as 0.37 at 45°. When the air gap decreases to 6 μm, the magnitude of the deflection beam dropped to a low level (<0.1), and the experimental results exhibit a high degree of agreement with the simulation results. The modulation depth for the deflection coefficient is defined as *MD* = (*D_max_* − *D_min_*)/*D_max_* (*D* is the value of deflection coefficient). By changing the air gap, a modulation depth of deflection coefficient as high as 89.3% at 0.6 THz is achieved. The effect of the air gap on the THz beam deflection capability is further verified by these experimental results.

Please note that the actual THz beam deflection efficiency may be higher than the measured results. The effective area of the fabricated device is only 7 × 7 mm^2^, which is much smaller than the area of the collimated terahertz wave (radius ~11 mm). Further optimization of the structural design and fabrication process to enlarge the effective area may improve the efficiency. In addition, the THz beam deflection efficiency of this device is still higher than that of previously reported metasurfaces with lossy dielectric materials, as illustrated in Table S4. This demonstrates that the large air gap design of metasurface is very effective in improving the THz beam steering efficiency.

**Fig. 6.**
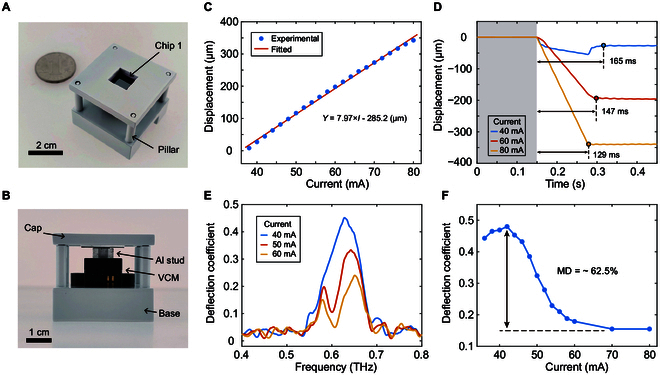
Measurement of the THz beam steering MTM. (A) Overview and (B) side view of the MTM (photograph). (C) Measured vertical displacement of the micro VCM versus applied currents. (D) Dynamic mechanical responses of the micro VCM. (E) Measured anomalous reflection spectra at the output angle of 44° for 3 different driving currents (*I* = 40, 50, 60 mA). (F) Measured deflection coefficient versus driving current (α = 44°). The modulation depth can reach approximately 62.5%.

To realize the continuous adjustment of the deflected beam amplitude, we utilized a micro VCM (TVF-8101KD; TDK, Japan) to carry the Chip 2 moving in the *Z* direction, as shown in Fig. S9. Figure 6A and B show the physical diagrams of the fabricated MTM for the manipulation of the terahertz beam. The Chip 1 was fixed at the center of the backside of the 3D printed cap, and the incident THz beam can arrive at the device through the square window (1 cm × 1 cm). A direct current supply was utilized to drive the micro VCM, and the vertical shift was measured by a spectral confocal microscope. Figure 6C illustrates that the vertical displacement (*Y*) exhibits a linear dependence on the applied current (*I*), which can be expressed as *Y* = 7.97× *I* − 285.2 (μm). The maximum displacement of the micro VCM with load was measured as approximately 342.4 μm at a driving current of 80 mA. Figure 6D shows the dynamic mechanical responses of the micro VCM in the assembled state for 3 different driving currents (*I* = 40, 60, and 80 mA). The response time for higher applied current (129 ms for 80 mA) is smaller than that for lower applied current (165 ms for 40 mA). Thus, the air gap between the Au ground plane and the metallic resonator array can be controlled with a fast switching time by applying driving currents to the micro VCM. When the applied current is 80 mA, the response time measured from 10% to 90% displacement is τ ≈ 100 ms. Thus, the 3 dB cutoff maximum modulation speed is approximately 3.5 Hz (*BW* = 0.35/τ) [43]. The rapid upward and downward movements of the Chip 2 driven by a square current signal are demonstrated in Movie S1 (Supplementary Materials).

The experimental setup shown in Fig. 5D was also employed to measure the THz beam deflection capability of the MTM. The fabricated MTM was fixed at the center of the rotation stage, and the THz receiver is fixed at an output angle of 44°. Figure 6E shows the measured deflection spectra ranging from 0.5 THz to 0.7 THz for *I* = 40, 50, and 60 mA. It can be seen that there is a certain frequency shift (~30 GHz) in the experimental and simulation results, which may be related to the assembly error. When the operating frequency is 0.63 THz, the measured deflection coefficient decreases from 0.48 to 0.18 as the applied current increases from 42 mA to 80 mA, as shown in Fig. 6F. The modulation depth of the deflection coefficient reached about 62.5% at the frequency of 0.63 THz. Consequently, the deflection coefficient can be tuned continuously by using the presented MTM on the basis of realizing beam steering function. Besides, the deflection efficiency of the THz beam can be increased by improving machining accuracy.

In the future, we aim to combine a large-stroke MEMS actuator and the metasurface to achieve a miniaturized tunable THz beam deflector, shrinking the overall size of the advanced THz system. Integrated fabrication of the 2 components will greatly reduce the errors generated in the assembly process, improving the efficiency of terahertz beam steering. Furthermore, constructing the demonstrated MTM as a large array structure can enrich the terahertz wave manipulation function, thereby expanding its potential for various applications such as THz communication, imaging, and space technology.

## Conclusion

In summary, we have developed an MTM for high-efficiency terahertz wave manipulation on the basis of changing the separation distance between the metallic resonator array and Au ground plane. By analyzing the EM responses of the designed air-spaced meta-atom based on the ECM, we uncovered that the phase span can cover 360° with high reflection coefficient (>0.95) when the air gap is sufficiently large (*g*_1_ = 50 μm). Then, we fabricated the silicon chips (Chip 1 and Chip 2) using the surface and bulk-micromachining processes. Measurement results show that the presented MTM can greatly improve the deflection coefficient to a remarkable maximum value of 0.60 for a deflection angle of 44.5° at 0.61 THz, agreeing well with the calculation by generalized Snell’s law. The deflection efficiency can be further improved by increasing the area of metasurface array and reducing the fabrication error. Moreover, we built a mechanically tunable THz metasurface, which consists of a metasurface layer (Chip 1), a gold-coated Si substrate (Chip 2), and a micro VCM. The air gap was varied by applying currents to the micro VCM, and thus, the deflection coefficient can be changed continuously. A deflection coefficient modulation depth of approximately 62.5% at the operating frequency is reached at a deflection angle of 44°. Our results have showcased the multifunctionality of the MTM, encompassing terahertz beam steering and continuous anomalous reflection coefficient regulation capabilities, achieving the multi-functional RIS. Our design provides a promising platform for realizing high-efficiency dynamic THz beam steering and modulation by utilizing the miniaturized meta-device.

## Data Availability

All data and findings of this study are available from the corresponding authors upon reasonable request.

## Supplementary Materials

Supplementary 1 Supplementary Materials are available from the Online Library or from the author. Figs. S1 to S9 Tables S1 to S4 Notes S1 to S12

## References

[1] Koenig S, Lopez-Diaz D, Antes J, Boes F, Henneberger R, Leuther A, Tessmann A, Schmogrow R, Hillerkuss D, Palmer R, et al. Wireless sub-THz communication system with high data rate. Nat Photonics. 2013;7(12):977–981.

[2] Ma ZT, Geng , Geng ZY, Fan ZY, Liu J, Chen HD. Modulators for terahertz communication: The current state of the art. Research. 2019;2019: 6482975. 31549075 10.34133/2019/6482975 PMC6750090

[3] Cui TJ, Liu S, Bai GD, Ma Q. Direct transmission of digital message via programmable coding metasurface. Research. 2019;2019: 2584509. 31549052 10.34133/2019/2584509 PMC6750087

[4] Stantchev RI, Yu X, Blu T, Pickwell-MacPherson E. Real-time terahertz imaging with a single-pixel detector. Nat Commun. 2020;11(1):2526. 32439984 10.1038/s41467-020-16370-x PMC7242476

[5] Wade CG, Šibalić N, de Melo NR, Kondo JM, Adams CS, Weatherill KJ. Real-time near-field terahertz imaging with atomic optical fluorescence. Nat Photonics. 2016;11(1):40–43.

[6] Fan K, Suen JY, Liu X, Padilla WJ. All-dielectric metasurface absorbers for uncooled terahertz imaging. Optica. 2017;4(6):601–604.

[7] Yamada T, Baron P, Neary L, Nishibori T, Larsson R, Kuroda T, Daerden F, Kasai Y. Observation capability of a ground-based terahertz radiometer for vertical profiles of oxygen and water abundances in martian atmosphere. IEEE Trans Geosci Remote Sens. 2022;60:1–11.

[8] Tang A, Reck T, Chattopadhyay G. CMOS system-on-chip techniques in millimeter-wave/THz instruments and communications for planetary exploration. IEEE Commun Mag. 2016;54(10):176–182.

[9] Yang X, Zhao X, Yang K, Liu Y, Liu Y, Fu W, Luo Y. Biomedical applications of terahertz spectroscopy and imaging. Trends Biotechnol. 2016;34(10):810–824. 27207226 10.1016/j.tibtech.2016.04.008

[10] Ahmadivand A, Gerislioglu B, Ramezani Z, Kaushik A, Manickam P, Ghoreishi SA. Functionalized terahertz plasmonic metasensors: Femtomolar-level detection of SARS-CoV-2 spike proteins. Biosens Bioelectron. 2021;177: Article 112971. 33434777 10.1016/j.bios.2021.112971 PMC7787065

[11] Tan XX, Zhong Y, Li RJ, Chang C. Neuromodulation of chemical synaptic transmission driven by THz photons. Research. 2022;2022:10. 10.34133/research.0010 PMC11404318 39285946

[12] Sizov F, Rogalski A. THz detectors. Prog Quantum Electron. 2010;34(5):278–347.

[13] Appleby R, Wallace HB. Standoff detection of weapons and contraband in the 100 GHz to 1 THz region. IEEE Trans Antennas Propag. 2007;55:2944–2956.

[14] Cai X, Tang R, Zhou H, Li Q, Ma S, Wang D, Liu T, Ling X, Tan W, He Q, et al. Dynamically controlling terahertz wavefronts with cascaded metasurfaces. Adv Photon. 2021;3(03): Article 036003.

[15] Ali A, Khalily M, Brown T, Tafazolli R. Metasurface-based THz reflectarray antenna with vortex multiplexing and beam-steering capabilities for future wireless communications. iScience. 2022;25(8): Article 104725. 35874095 10.1016/j.isci.2022.104725 PMC9304595

[16] Cheng J, Dong X, Chen S, Yuan Y, Wen Q, Chang S. Terahertz metagrating accordion for dynamic beam steering. Adv Opt Mater. 2022;10(11):2200008.

[17] Zhang Z, Yan X, Liang L, Wei D, Wang M, Wang Y, Yao J. The novel hybrid metal-graphene metasurfaces for broadband focusing and beam-steering in farfield at the terahertz frequencies. Carbon. 2018;132:529–538.

[18] Alonso-delPino M, Jung-Kubiak C, Reck T, Llombart N, Chattopadhyay G. Beam scanning of silicon lens antennas using integrated piezomotors at submillimeter wavelengths. IEEE Trans Terahertz Sci Technol. 2019;9(1):47–54.

[19] Gulkis S, Frerking MA, Crovisier J, Beaudin G. MIRO: Microwave instrument for rosetta orbiter. Space Sci Rev. 2006;128(1-4):561–597.

[20] Yang Y, Gurbuz OD, Rebeiz GM. An eight-element 370–410-GHz phased-array transmitter in 45-nm CMOS SOI with peak EIRP of 8–8.5 dBm. IEEE Trans Microw Theory Tech. 2016;64(12):4241–4249.

[21] Hu Z, Wang C, Han R. A 32-unit 240-GHz heterodyne receiver array in 65-nm CMOS with array-wide phase locking. IEEE J Solid State Circuits. 2019;54(5):1216–1227.

[22] Monnai Y, Altmann K, Jansen C, Hillmer H, Koch M, Shinoda H. Terahertz beam steering and variable focusing using programmable diffraction gratings. Opt Express. 2013;21(2):2347–2354. 23389214 10.1364/OE.21.002347

[23] Ren FF, Gu JX, Wei H, Xu GP, Zhao JP, Dou SL, Li Y. Effect of unit cell shape on switchable infrared metamaterial VO2 absorbers/emitters. Research. 2021;2021: 9804183. 33982002 10.34133/2021/9804183 PMC8087995

[24] Wen YZ, Zhou J. Artificial generation of high harmonics via nonrelativistic Thomson scattering in metamaterial. i. 2019;2019: 8959285. 10.34133/2019/8959285 PMC6750065 31549093

[25] Fan YC, He X, Zhang F, Cai W, Li C, Fu Q, Sydorchuk NV, Prosvirnin SL. Fano-resonant hybrid metamaterial for enhanced nonlinear tunability and hysteresis behavior. Research. 2021;2021:9754083. 34485916 10.34133/2021/9754083 PMC8380421

[26] Yu NF, Genevet P, Kats MA, Aieta F, Tetienne JP, Capasso F, Gaburro Z. Light propagation with phase discontinuities: Generalized laws of reflection and refraction. Science. 2011;334:333–337. 21885733 10.1126/science.1210713

[27] Bai XD, Zhang FL, Sun L, Cao AJ, Zhang J, He C, Liu LH, Jianquan Y, Zhu WR. Time-modulated transmissive programmable metasurface for low sidelobe beam scanning. Research. 2022;2022:9825903. 35928303 10.34133/2022/9825903 PMC9297726

[28] Zhang L, Cui TJ. Space-time-coding digital metasurfaces: Principles and applications. Research. 2021;2021:9802673. 34386772 10.34133/2021/9802673 PMC8328401

[29] Fu X, Yang F, Liu C, Wu X, Cui TJ. Terahertz beam steering technologies: From phased arrays to field-programmable metasurfaces. Adv Opt Mater. 2019;8:1900628.

[30] Shrekenhamer D, Chen W-C, Padilla WJ. Liquid crystal tunable metamaterial absorber. Phys Rev Lett. 2013;110(17): Article 177403. 23679774 10.1103/PhysRevLett.110.177403

[31] Nemati A, Wang Q, Hong M, Teng J. Tunable and reconfigurable metasurfaces and metadevices. Opto-Electron Adv. 2018;1(5):18000901–18000925.

[32] He Q, Sun SL, Zhou L. Tunable/reconfigurable metasurfaces: Physics and applications. Research. 2019;2019:1849272. 31549047 10.34133/2019/1849272 PMC6750114

[33] Tao H, Strikwerda AC, Fan K, Padilla WJ, Zhang X, Averitt RD. Reconfigurable terahertz metamaterials. Phys Rev Lett. 2009;103(14): Article 147401. 19905602 10.1103/PhysRevLett.103.147401

[34] Pitchappa P, Ho CP, Dhakar L, Lee C. Microelectromechanically reconfigurable interpixelated metamaterial for independent tuning of multiple resonances at terahertz spectral region. Optica. 2015;2(6):571–578.

[35] Zhao X, Schalch J, Zhang J, Seren HR, Duan G, Averitt RD, Zhang X. Electromechanically tunable metasurface transmission waveplate at terahertz frequencies. Optica. 2018;5(3):303–310.

[36] Zhao X, Sun Z, Zhang L, Wang Z, Xie R, Zhao J, You R, You Z. Review on metasurfaces: An alternative approach to advanced devices and instruments. Adv Dev Instrum. 2022;2022:9765089.

[37] Zhao X, Duan G, Li A, Chen C, Zhang X. Integrating microsystems with metamaterials towards metadevices. Microsyst Nanoeng. 2019;5:5. 31057932 10.1038/s41378-018-0042-1 PMC6348284

[38] Holsteen AL, Cihan AF, Brongersma ML. Temporal color mixing and dynamic beam shaping with silicon metasurfaces. Science. 2019;365(6450):257–260. 31320534 10.1126/science.aax5961

[39] Meng C, Thrane PCV, Ding F, Gjessing J, Thomaschewski M, Wu C, Dirdal C, Bozhevolnyi SI. Dynamic piezoelectric MEMS-based optical metasurfaces. Sci Adv. 2021;7(26):eabg5639. 34162551 10.1126/sciadv.abg5639 PMC8221626

[40] Zhuang X, Zhang W, Wang K, Gu Y, An Y, Zhang X, Gu J, Luo D, Han J, Zhang W. Active terahertz beam steering based on mechanical deformation of liquid crystal elastomer metasurface. Light Sci Appl. 2023;12(1):14. 36596761 10.1038/s41377-022-01046-6 PMC9810742

[41] Duan G, Schalch J, Zhao X, Zhang J, Averitt RD, Zhang X. An air-spaced terahertz metamaterial perfect absorber. Sens Actuator A Phys. 2018;280:303–308.

[42] Sun Z, Zhao X, Zhang L, Mei Z, Zhong H, You R, Lu W, You Z, Zhao J. WiFi energy-harvesting antenna inspired by the resonant magnetic dipole metamaterial. Sensors. 2022;22(17):6523. 36080982 10.3390/s22176523 PMC9460457

[43] Liu MK, Susli M, Silva D, Putrino G, Kala H, Fan S, Cole M, Faraone L, Wallace VP, Padilla WJ, et al. Ultrathin tunable terahertz absorber based on MEMS-driven metamaterial. Microsyst Nanoeng. 2017;3:17033. 31057871 10.1038/micronano.2017.33 PMC6445006

