# Supplementary material for: High-Efficiency Dynamic Terahertz Deflector Utilizing a Mechanically Tunable Metasurface
Source: Research (Wash D C). 2023 Dec 1;6:0274. doi: 10.34133/research.0274 (PMC10907017; doi:10.34133/research.0274)
Supplement: Supplementary 1 — Supplementary Materials are available from the Online Library or from the author. Figs. S1 to S9 Tables S1 to S4 Notes S1 to S12 [file research.0274.f1.docx]

**Supplementary Materials**

**High-Efficiency Dynamic Terahertz Deflector Utilizing a Mechanically Tunable Metasurface**

Zhenci Sun^1,2,3,#^, Chao Liang^1,2,3,#^, Chen Chen^1^, Xiayu Wang^1,2,3^, Enze Zhou^1,2,3^, Xiaomeng Bian^4^, Yuanmu Yang^1^, Rui You^4,*^, Xiaoguang Zhao^1,2,3,*^, Jiahao Zhao^1,2,3,*^, and Zheng You^1,2,3^

^1^ Department of Precision Instrument, Tsinghua University, Beijing 100084, China

^2^ State Key Laboratory of Precision Measurement Technology and Instrument, Tsinghua University, Beijing 100084, China

^3^ Beijing Advanced Innovation Center for Integrated Circuits Beijing, 100084, China

^4^ School of Instrument Science and Opto-Electronic Engineering, Beijing Information Science and Technology University, Beijing 100016, China

^#^ Zhenci Sun and Chao Liang contributed equally to this work.

* Correspondence:

yourui@bistu.edu.cn, Rui You;

zhaoxg@mail.tsinghua.edu.cn, Xiaoguang Zhao;

falxon@mail.tsinghua.edu.cn, Jiahao Zhao.

1. **Effects of the air gap on beam steering function.**

When the air gap between the metallic resonator array and Au ground plane is equivalent to the optimal design value, six air-spaced meta-atoms with uniform phase gradient (~ π/3) form a Huygens’ subarray, and the reflected THz beam will deflect at an output angle of α = 45° according to the generalized Snell’s law [S1], as shown in Fig. S1A. When the air gap is decreased to about 0 μm, the vertically incident THz beam will be specular reflected, and the deflected coefficient of the angle (α) is greatly reduced due to the disappearance of phase discontinuities, as shown in Fig. S1B.


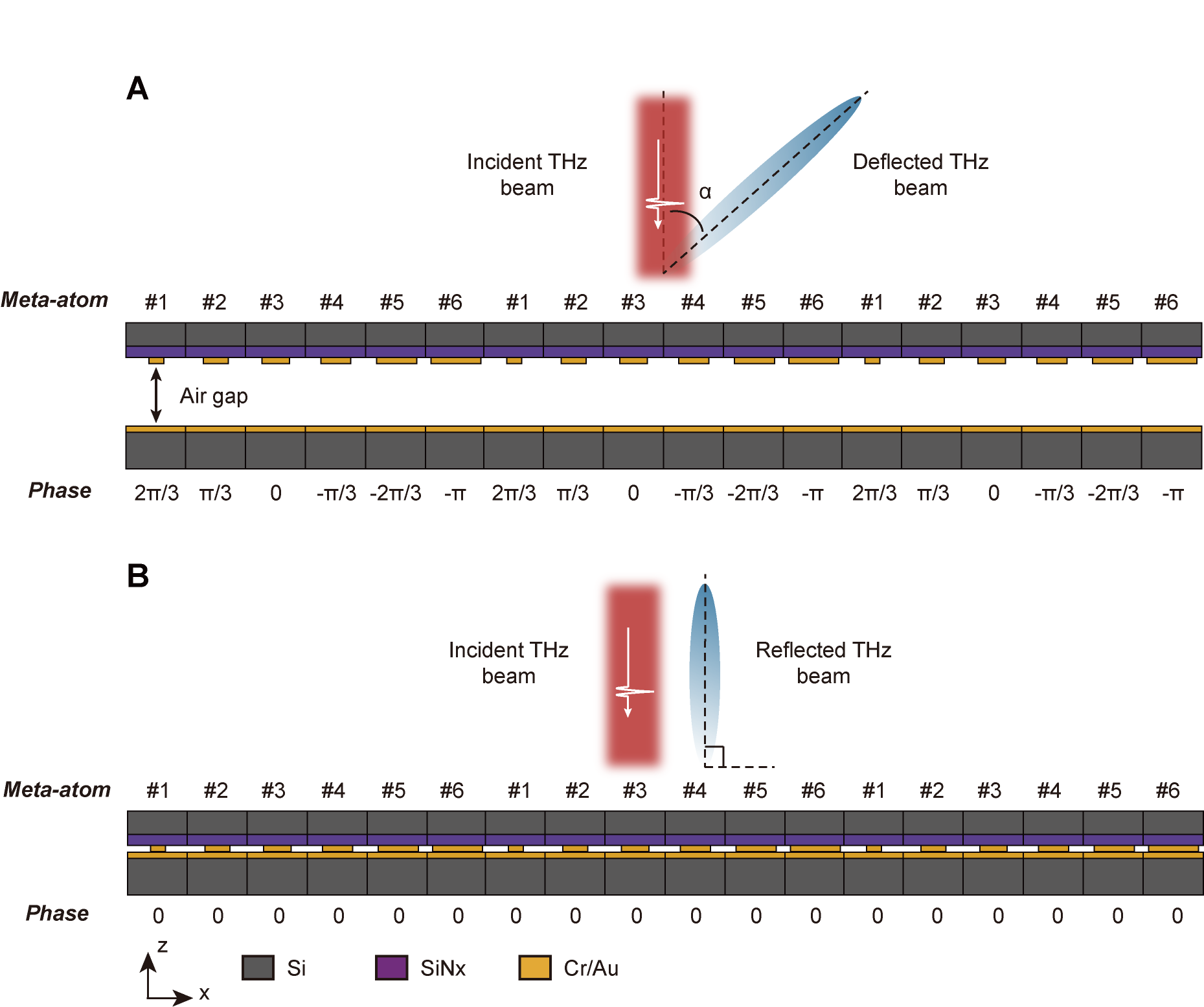


**Fig. S1. Schematic diagrams of THz beam manipulation for the designed air-spaced metasurface with different air gaps.** **(A)** equivalent to the optimal design value, and **(B)** close to zero.

1. **Thickness values of different structural layers.**

In the simulation model, thickness values of different structural layers in the designed meta-atom are listed in Table S1.

Table S1 Thickness values of different structural layers

| Parameters | Symbol | Value |
| --- | --- | --- |
| Thickness of HR silicon | *t_1_* | 60 μm |
| Thickness of silicon nitride | *t_2_* | 0.2 μm |
| Thickness of metallic resonator | *t_3_* | 0.15 μm |
| Thickness of Au ground plane | *t_4_* | 0.15 μm |

1. **Derivation of the reflection coefficient (*r*) for the meta-atom.**

For the top metallic resonator, the impedance (*Z_1_*) with a serially connected resistor (*R_1_*), inductor (*L_1_*), and capacitor (*C_1_*) can be expressed as:

$$Z_{1}=R_{1}+i\omega L_{1}+\frac{1}{i\omega C_{1}} (S1)$$

where $\omega$ is the operation frequency in radians. A paralleled impedance (*Z_2_*) represents the interlayer coupling induced by the air gap, which can be described as [S2]:

$$Z_{2}=i\sqrt{{\mu_{0}}/{\varepsilon_{0}}}tan\left( \beta g_{1} \right)=i\sqrt{{\mu_{0}}/{\varepsilon_{0}}}tan\left( \frac{\omega\times g_{1}}{c} \right) (S2)$$

where $c$ is the velocity of light, $g_{1}$ is the air gap between the metallic resonator array and the Au ground plane, $\mu_{0}$ and $\varepsilon_{0}$ are the permeability and permittivity of the vacuum, respectively.

The overall input impedance (*Z_in_*) of the meta-atom can be derived by combining the above equations, that is:

$$Z_{in}=Z_{1}{//Z}_{2}=\frac{\left( R_{1}+i\omega L_{1}+\frac{1}{i\omega C_{1}} \right)\times i\sqrt{{\mu_{0}}/{\varepsilon_{0}}}tan\left( \frac{\omega\times g_{1}}{c} \right)}{\left( R_{1}+i\omega L_{1}+\frac{1}{i\omega C_{1}} \right)+i\sqrt{{\mu_{0}}/{\varepsilon_{0}}}tan\left( \frac{\omega\times g_{1}}{c} \right)} (S3)$$

The reflection coefficient (*r*) of the meta-atom can be calculated from the impedance mismatch [S3]:

$$r=\frac{Z_{in}-Z_{0}}{Z_{in}+Z_{0}}=\frac{\frac{\left( R_{1}+i\omega L_{1}+\frac{1}{i\omega C_{1}} \right)\times i\sqrt{{\mu_{0}}/{\varepsilon_{0}}}tan\left( \frac{\omega\times g_{1}}{c} \right)}{\left( R_{1}+i\omega L_{1}+\frac{1}{i\omega C_{1}} \right)+i\sqrt{{\mu_{0}}/{\varepsilon_{0}}}tan\left( \frac{\omega\times g_{1}}{c} \right)}-Z_{0}}{\frac{\left( R_{1}+i\omega L_{1}+\frac{1}{i\omega C_{1}} \right)\times i\sqrt{{\mu_{0}}/{\varepsilon_{0}}}tan\left( \frac{\omega\times g_{1}}{c} \right)}{\left( R_{1}+i\omega L_{1}+\frac{1}{i\omega C_{1}} \right)+i\sqrt{{\mu_{0}}/{\varepsilon_{0}}}tan\left( \frac{\omega\times g_{1}}{c} \right)}+Z_{0}}$$

$$=\frac{\left( R_{1}+i\omega L_{1}+\frac{1}{i\omega C_{1}} \right)\times i\sqrt{{\mu_{0}}/{\varepsilon_{0}}}tan\left( \frac{\omega\times g_{1}}{c} \right)-Z_{0}\times\left[ \left( R_{1}+i\omega L_{1}+\frac{1}{i\omega C_{1}} \right)+i\sqrt{{\mu_{0}}/{\varepsilon_{0}}}tan\left( \frac{\omega\times g_{1}}{c} \right) \right]}{\left( R_{1}+i\omega L_{1}+\frac{1}{i\omega C_{1}} \right)\times i\sqrt{{\mu_{0}}/{\varepsilon_{0}}}tan\left( \frac{\omega\times g_{1}}{c} \right)+Z_{0}\times\left[ \left( R_{1}+i\omega L_{1}+\frac{1}{i\omega C_{1}} \right)+i\sqrt{{\mu_{0}}/{\varepsilon_{0}}}tan\left( \frac{\omega\times g_{1}}{c} \right) \right]}$$

$$(S4)$$

where $Z_{0}$ is the wave impedance of vacuum. Then, we can multiply the numerator (*A*) and denominator (*B*) in Eq. (S4) by $i\omega C_{1}$, as following:

$$A=-\omega R_{1}C_{1}\sqrt{{\mu_{0}}/{\varepsilon_{0}}}tan\left( \frac{\omega\times g_{1}}{c} \right)-i\omega^{2}L_{1}C_{1}\sqrt{{\mu_{0}}/{\varepsilon_{0}}}tan\left( \frac{\omega\times g_{1}}{c} \right)+i\sqrt{{\mu_{0}}/{\varepsilon_{0}}}tan\left( \frac{\omega\times g_{1}}{c} \right)-i\omega R_{1}C_{1}Z_{0}+\omega^{2}L_{1}C_{1}Z_{0}-Z_{0}+\omega C_{1}Z_{0}\sqrt{{\mu_{0}}/{\varepsilon_{0}}}tan\left( \frac{\omega\times g_{1}}{c} \right) (S5)$$

$$B=-\omega R_{1}C_{1}\sqrt{{\mu_{0}}/{\varepsilon_{0}}}tan\left( \frac{\omega\times g_{1}}{c} \right)-i\omega^{2}L_{1}C_{1}\sqrt{{\mu_{0}}/{\varepsilon_{0}}}tan\left( \frac{\omega\times g_{1}}{c} \right)+i\sqrt{{\mu_{0}}/{\varepsilon_{0}}}tan\left( \frac{\omega\times g_{1}}{c} \right)+i\omega R_{1}C_{1}Z_{0}-\omega^{2}L_{1}C_{1}Z_{0}+Z_{0}-\omega C_{1}Z_{0}\sqrt{{\mu_{0}}/{\varepsilon_{0}}}tan\left( \frac{\omega\times g_{1}}{c} \right) (S6)$$

Combining Eqs. (S5) and (S6), we can obtain Eq. (1) in the manuscript.

1. **The retrieved parameters in the equivalent circuit model for six meta-atoms.**

The air gap of the six meta-atoms is 50 μm, and thus the paralleled impedence *Z_2_* can be described as $i377\times tan\left( \omega\times1.6667\times{10}^{-13} \right)$ by Eq. (S2). $\omega$ represents the angular frequency. By fitting the simulated and calculated results quantitatively, we can obtain three proper ECM parameters of six meta-atoms, including *R_1_*, *L_1_*, and *C_1_*, as listed in Table S2.

Table S2 Quantitative fitting ECM parameters of six meta-atoms

| Meta-atom | *R_1_* (Ω) | *L_1_* (×10^-9^ H) | *C_1_* (×10^-16^ F) |
| --- | --- | --- | --- |
| #1 | 0.30 | 0.18 | 1.90 |
| #2 | 2.76 | 0.25 | 2.05 |
| #3 | 2.65 | 0.24 | 2.32 |
| #4 | 2.61 | 0.23 | 2.57 |
| #5 | 2.10 | 0.21 | 3.08 |
| #6 | 1.06 | 0.11 | 7.08 |

As the Table S2 shows, the capacitance *C_1_* increases as the gap between the contiguous metallic resonator decreases. The changes in *R_1_* and *L_1_* are much smaller than that in *C_1_*, meaning that the capacitance variation is the main reason for the tunning of amplitude and phase responses. Therefore, the fitting results manifest the underlying physics of the meta-atom design.

1. **Numerical simulation of the electromagnetic responses of the meta-atoms in subarray with two different air gaps.**

This section provides more details about simulated results of the EM properties of the selected air-spaced meta-atoms with two different air gaps (6 μm and 100 μm) at the operating frequency of 0.6 THz. When *g_1_* is set to be 6 μm, the reflection coefficient (blue line) and phase responses (red line) of #1~#5 meta-atoms are nearly consistent except for the #6 meta-atom, as shown in Fig. S2A. The phase difference between #6 meta-atom and the first five is only about 100°, and the phase coverage cannot achieve 360°. When *g_1_* is 100 μm, the maximum value of the phase gradient between the adjacent meta-atoms can reach 122.9° (#1 and #2), and the minimum value is about 30° (#3 and #4), as shown in Fig. S2B. Thus, the phase responses of the six meta-atoms in subarray is not linear.


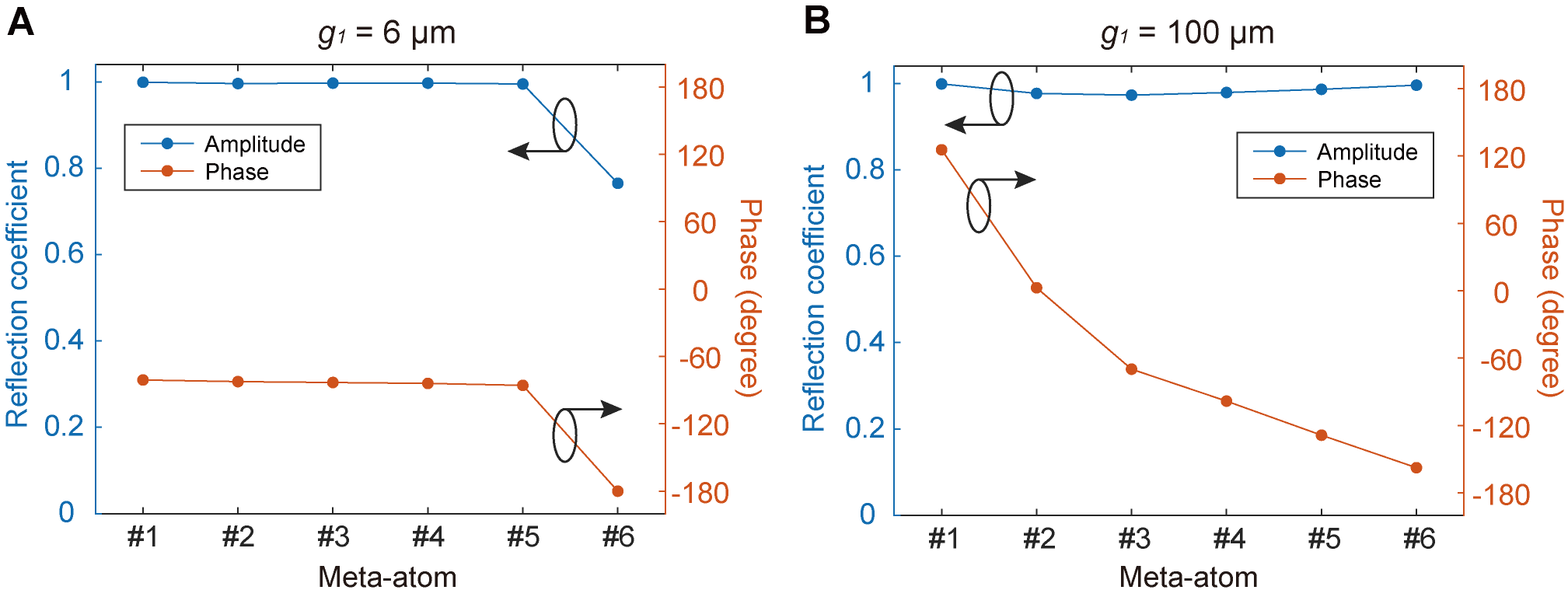


**Fig. S2. Simulated EM properties of six selected meta-atoms.** **(A)** *g_1_* = 6 μm, and **(B)** *g_1_* = 100 μm.

In addition, we calculated the phase gradients of six air-spaced meta-atoms with three different air gaps at the operating frequency of 0.6 THz, as listed in Table S3. The phase gradient is defined as ${\Delta\varphi}_{i}=\varphi_{i}-\varphi_{i+1}$, in which $\varphi_{i}$ is the reflection phase response of the *i*th meta-atom. As can be seen from the calculation results, the phase gradients at 0.6 THz are not constant after air gap tuning.

Table S3 Phase gradients of the adjacent air-spaced meta-atoms at 0.6 THz.

| ${\Delta\varphi}_{i}$ | *g_1_* = 6 μm | *g_1_* = 50 μm | *g_1_* = 100 μm |
| --- | --- | --- | --- |
| *i* = 1 | 1.5° | 59.0° | 122.9° |
| *i* = 2 | 0.8° | 60.9° | 72.5° |
| *i* = 3 | 0.8° | 56.8° | 28.5° |
| *i* = 4 | 1.7° | 67.1° | 30.4° |
| *i* = 5 | 94.3° | 56.2° | 30.0° |

1. **Effects of the air gap on THz beam of different diffraction orders.**

The effects of the air gap on the terahertz beams at different diffraction orders have been investigated through full-wave simulation at the operating frequency of 0.6 THz (Figs. S3, A to H). The E-field amplitudes (diffraction coefficient) at different diffraction orders (m = 0 and ±1) are different by tunning air gap. Fig. S3I illustrates the diffraction coefficients of different orders (m = 0 and ±1) as a function of the air gap (*g_1_*) for the incident terahertz wave at 0.6 THz.


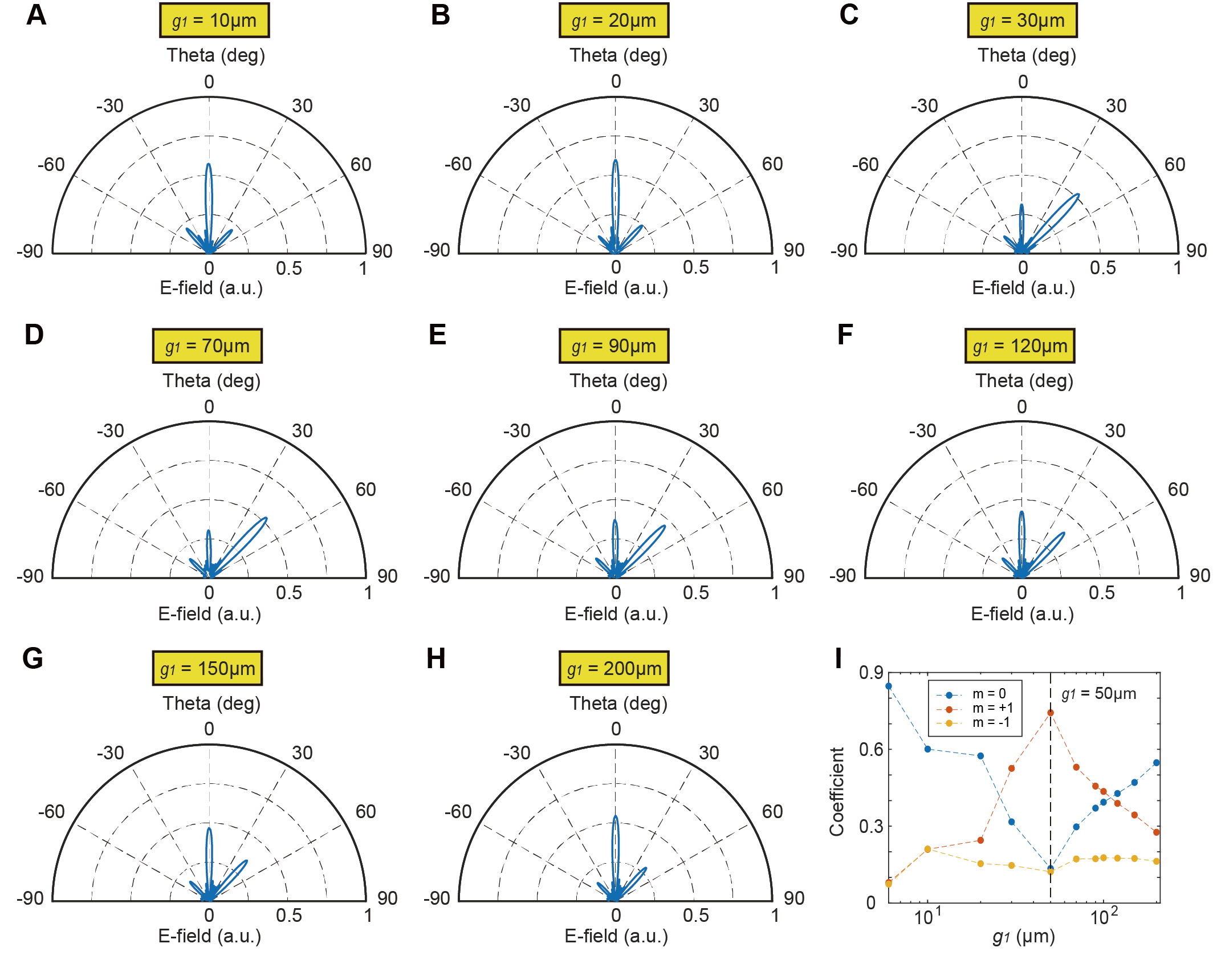


**Fig. S3. Simulated far-field scattering patterns of the metasurface array with different air gaps. (A)** *g_1_* = 10 μm, **(B)** *g_1_* = 20 μm, **(C)** *g_1_* = 30 μm, **(D)** *g_1_* = 70 μm, **(E)** *g_1_* = 90 μm, **(F)** *g_1_* = 120 μm, **(G)** *g_1_* = 150 μm, and **(H)** *g_1_* = 200 μm. **(I)** Diffraction coefficient of different orders (m = 0, ±1) calculated as a function of the air gap for the normal incident terahertz wave at 0.6 THz.

As the air gap increases from 6 to 200 μm, the change trends of diffraction coefficient at 0 order (blue dot dashed line) and +1 order (red dot dashed line) are opposite, while the diffraction coefficient at -1 order (yellow dot dashed line) basically remains below 0.2, and its change is not obvious. Thus, the diffraction coefficient at 0 and +1 orders can be considered as interacting. The increase of the diffraction coefficient (m = 0) is accompanied by the decrease of the diffraction coefficient (m = +1). Thus, we can change the deflection coefficient continuously by controlling the air gap.

1. **KOH wet etching of the first silicon wafer.**

Fig. S4A shows the photograph of wet etched first silicon wafer (4 inch). It can be seen that the traces are designed around each small square chips. We can dice the 4-inch silicon wafer into square chips along the etched traces, and the side length of Chip 1 is approximately 1.7 cm, as shown in Fig. S4B.


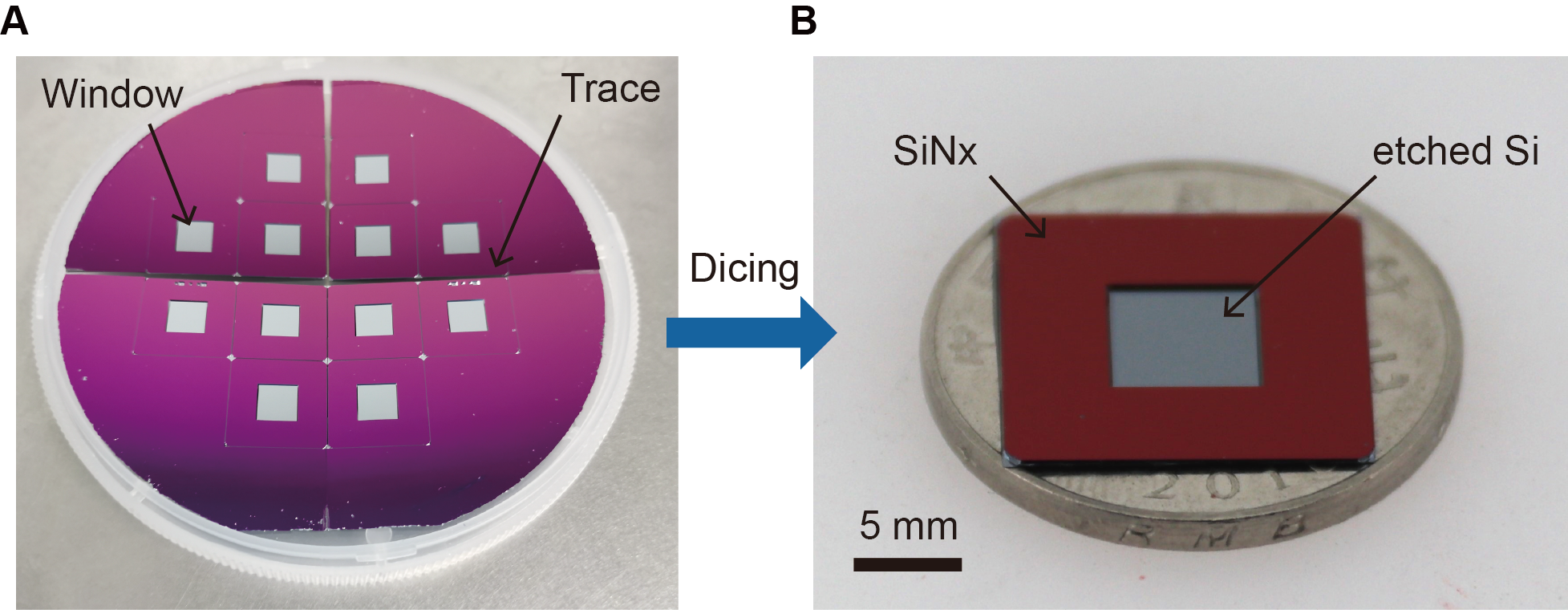


**Fig. S4. Photograph of the wet etched silicon wafer. (A)** 4-inch HR silicon wafer (> 10^4^ Ω·cm), **(B)** Chip 1 (after dicing).

A 33wt% potassium hydroxide (KOH) solution was used as corrosion fluids, and the wet etching rate was about 1 μm/min. After KOH wet etching, a step structure was formed between the SiNx membrane (top layer) and etched Si surface (bottom layer). Fig. S5 illustrates the surface morphology of the step structure after wet etching characterized by 3D optical profilometer (ContourGT; Bruker, Germany). The initial thickness of the first silicon wafer and the etching depth of the step structure were measured as approximately 324 μm and 264 μm, respectively. Thus, the thickness of remaining structural layer was calculated as about 60 μm.


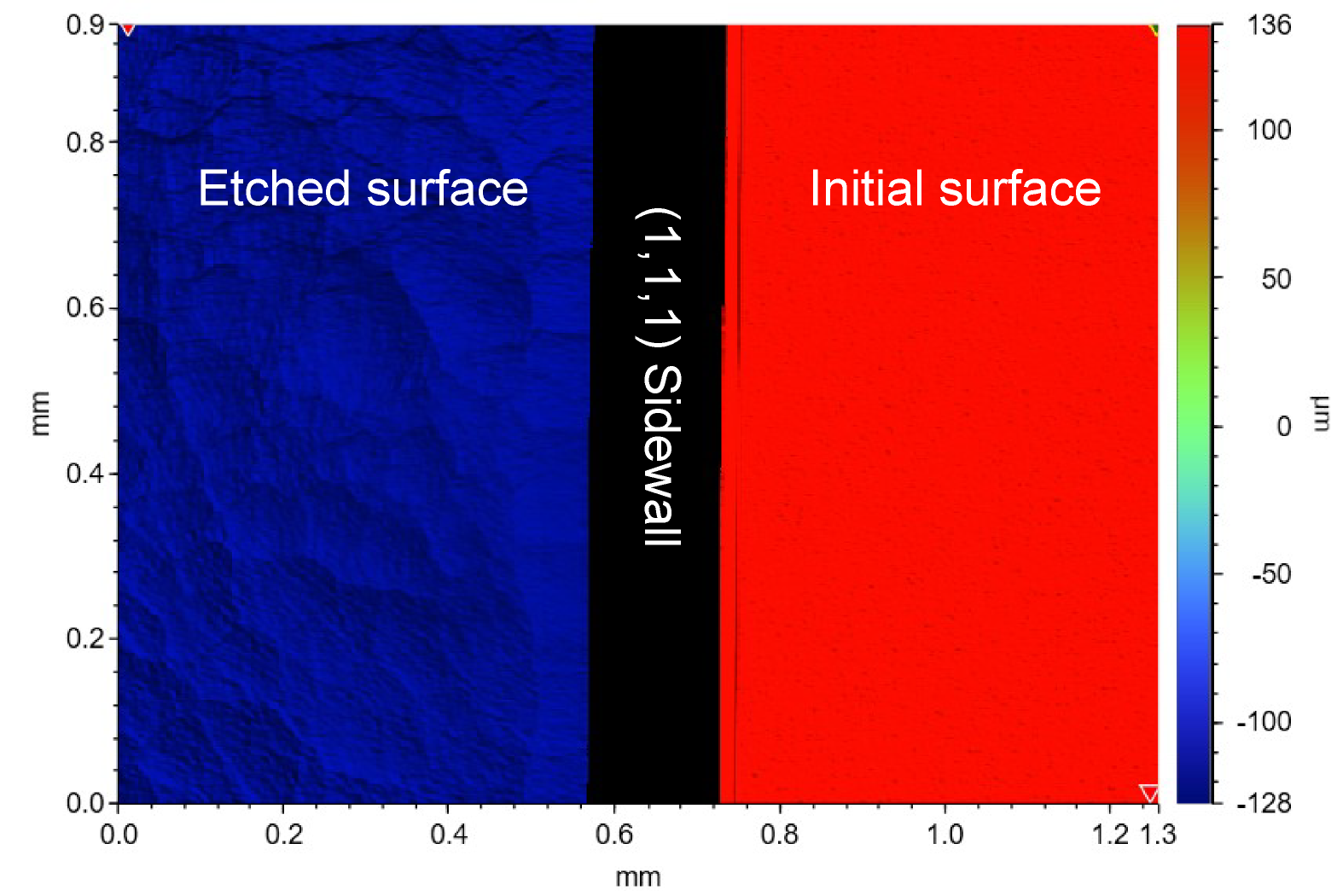


**Fig. S5.** Measured surface morphology of the step structure.

1. **The bonding of the two silicon chips.**

To bond the Chip 1 and Chip 2 with different air gaps, we used several ultra-thin double-sided sticky spacers with different thickness (6 μm, 50 μm, and 100 μm). Fig. S6A illustrates that a patterned adhesive spacer was attached to the upper surface of the Au ground plane. The adhesive spacer should be on the edge to avoid contacting with the central metallic resonator array of Chip 1. Then, the Chip 1 was combined with Chip 2 by flip-chip bonding, as shown in Fig. S6B.


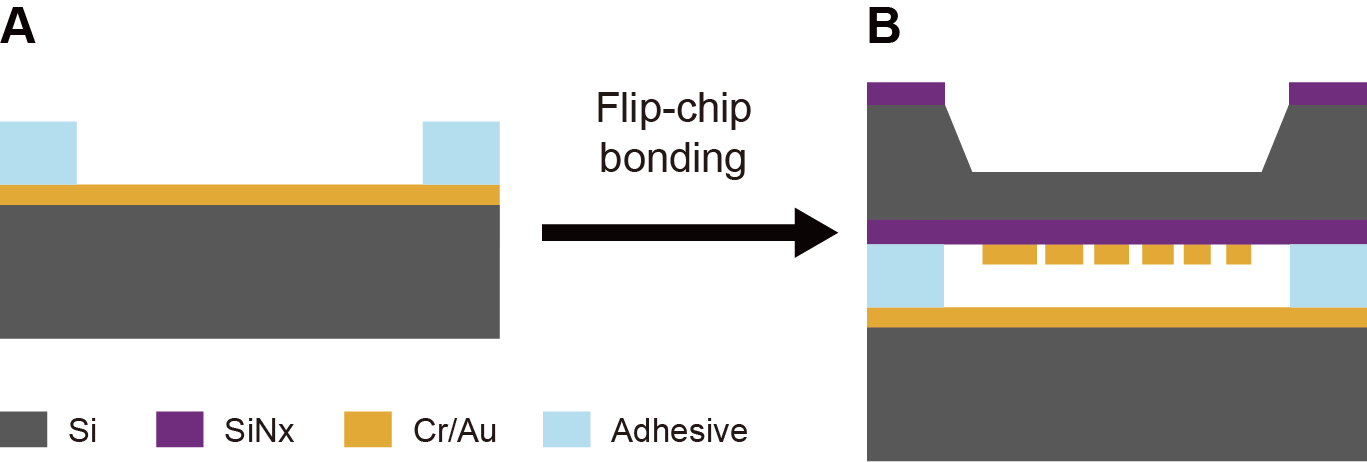


**Fig. S6.** Flip-chip bonding of the air-spaced metasurface.

1. **SEM images of the air gap.**

The cross-sectional SEM images of the air gap between the two silicon chips are shown in Fig. S7. The air gaps shown in Fig. S7A and Fig. S7B were measured as approximately 6.1 μm and 98.3 μm, respectively. It can be seen that there are slight manufacturing deviations.


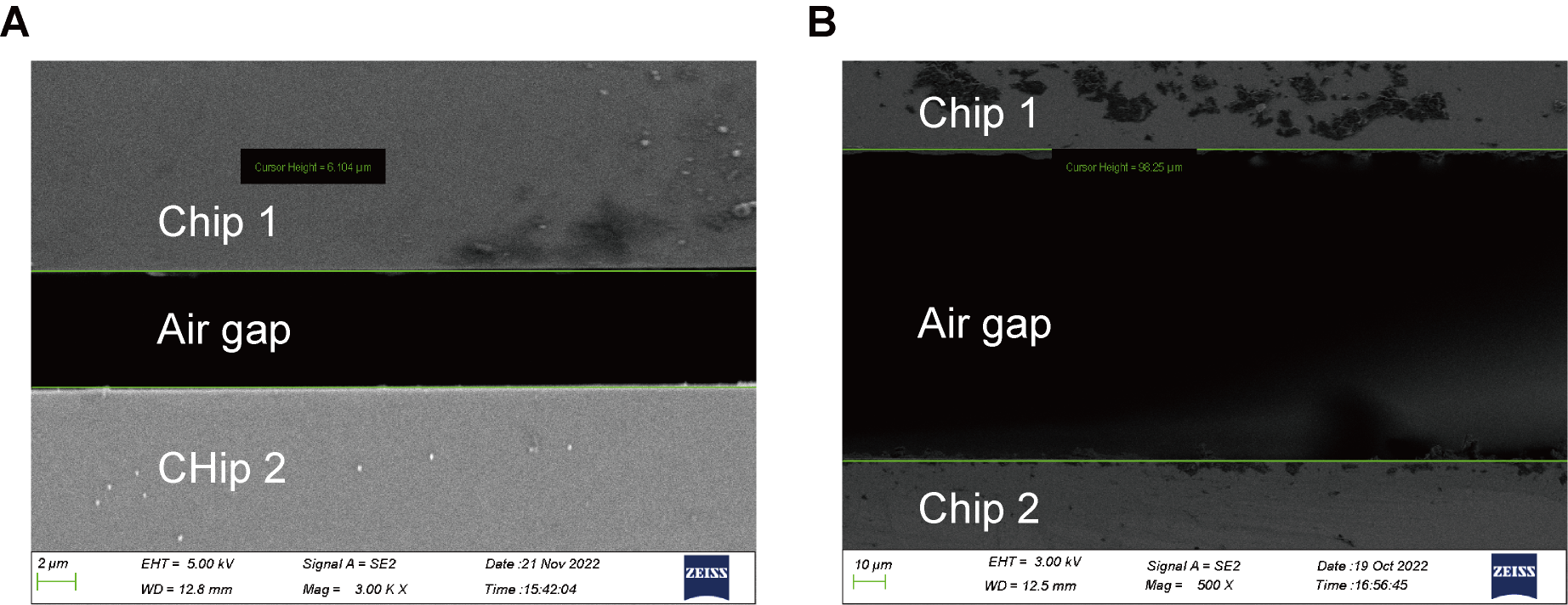


**Fig. S7. SEM images of the air gap between two silicon chips. (A)** *g_1_* = 6.1 μm, **(B)** *g_1_* = 98.3 μm.

1. **Specific measurement and calculation of reflection coefficient.**

Firstly, we measured the terahertz time-domain signal of the air without devices as the reference signal, as shown in Fig. S8A. The received time domain signal is the voltage signal between the two electrodes of the photoconductive antenna, proportional to the electric field strength of the THz wave. The pulse length of the transmitted THz signal is set to 200 ps.


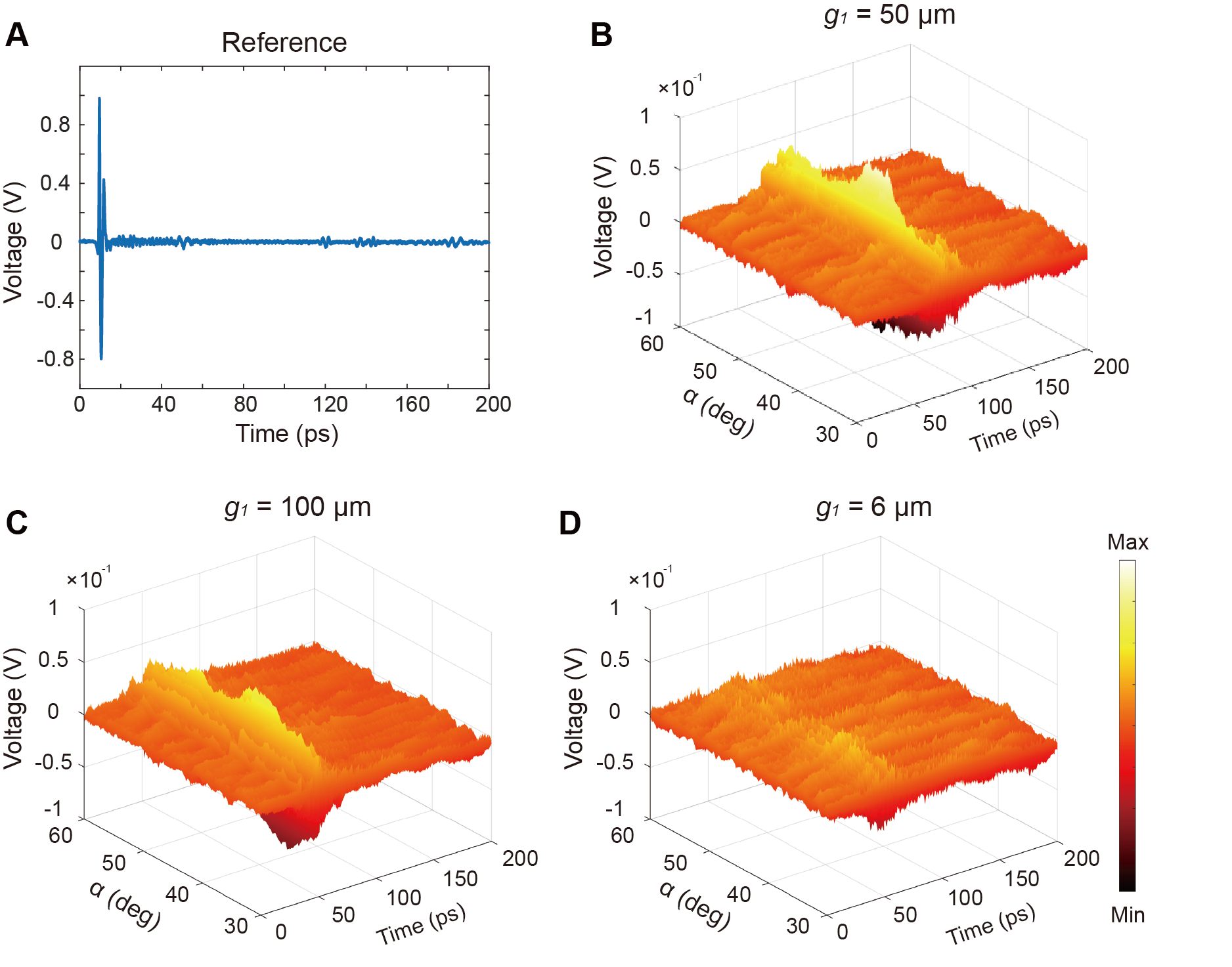


**Fig. S8. Measured original signals. (A)** the time-domain signal of the air without devices. Received time-domain signals at varied output angles: **(B)** *g_1_* = 50 μm, **(C)** *g_1_* = 100 μm, and **(D)** *g_1_* = 6 μm.

The measured time-domain signal of air was then converted to frequency-domain curve (reference spectrum) by using Fast fourier transform (FFT). Next, the devices with three different air gaps (6, 50, and 100 μm) were sequentially fixed at the center of the rotation stage. The received time-domain signals at varied deflection angles (30~60°) were shown in Figs. S8B-S8D. FFT was applied to the measured time-domain signals to obtain the frequency spectra. Finally, the frequency spectra of devices were normalized with the reference spectrum (plotted in Fig. S8A) to calculate the reflection coefficient of three devices.

1. **Comparison of properties for various THz beam steering metasurfaces.**

Table S4 Performances of recently reported THz beam steering metasurfaces

| Ref. | Year | Tunable methods | Deflection amplitude | Deflection efficiency |
| --- | --- | --- | --- | --- |
| [S4] | 2015 | Semiconductor | ~ 0.40 | ~ 16.0% |
| [S5] | 2018 | Semiconductor | ~ 0.35 | ~ 12.3% |
| [S6] | 2019 | / | ~ 0.30 | ~ 9.0% |
| [S7] | 2020 | Liquid crystal | ~ 0.31 | ~ 9.6% |
| [S8] | 2020 | Liquid crystal | ~ 0.20 | ~ 4.0% |
| [S9] | 2022 | Liquid crystal | ~ 0.15 | 2.3% |
| [S10] | 2022 | Vanadium dioxide (VO2) | ~ 0.27 | ~ 7.3% |
| [S11] | 2023 | Liquid crystal elastomer | < 0.30 | < 9.0% |
| This work | 2023 | MEMS | ~ 0.60 | ~ 36.0% |

The performance of the presented THz beam steering metasurface in this paper is compared with previously published works, as shown in Table S4. The deflection efficiency is equal to the square of deflection amplitude, which means the ratio of THz beam energy. Based on the data presented in the table, the THz beam deflection efficiency of the proposed device is much higher than that of other previous works. It can be attributed to the removal of lossy dielectric materials.

1. **Assembly of the MTM for THz beam steering.**

Fig. S9 illustrates the photo image of the assembled THz beam steering MTM. The micro VCM was fixed on the 3D printed base by two hexagon bolts. The Si substrate (Chip 2) was connected to the micro VCM by a solid stud.


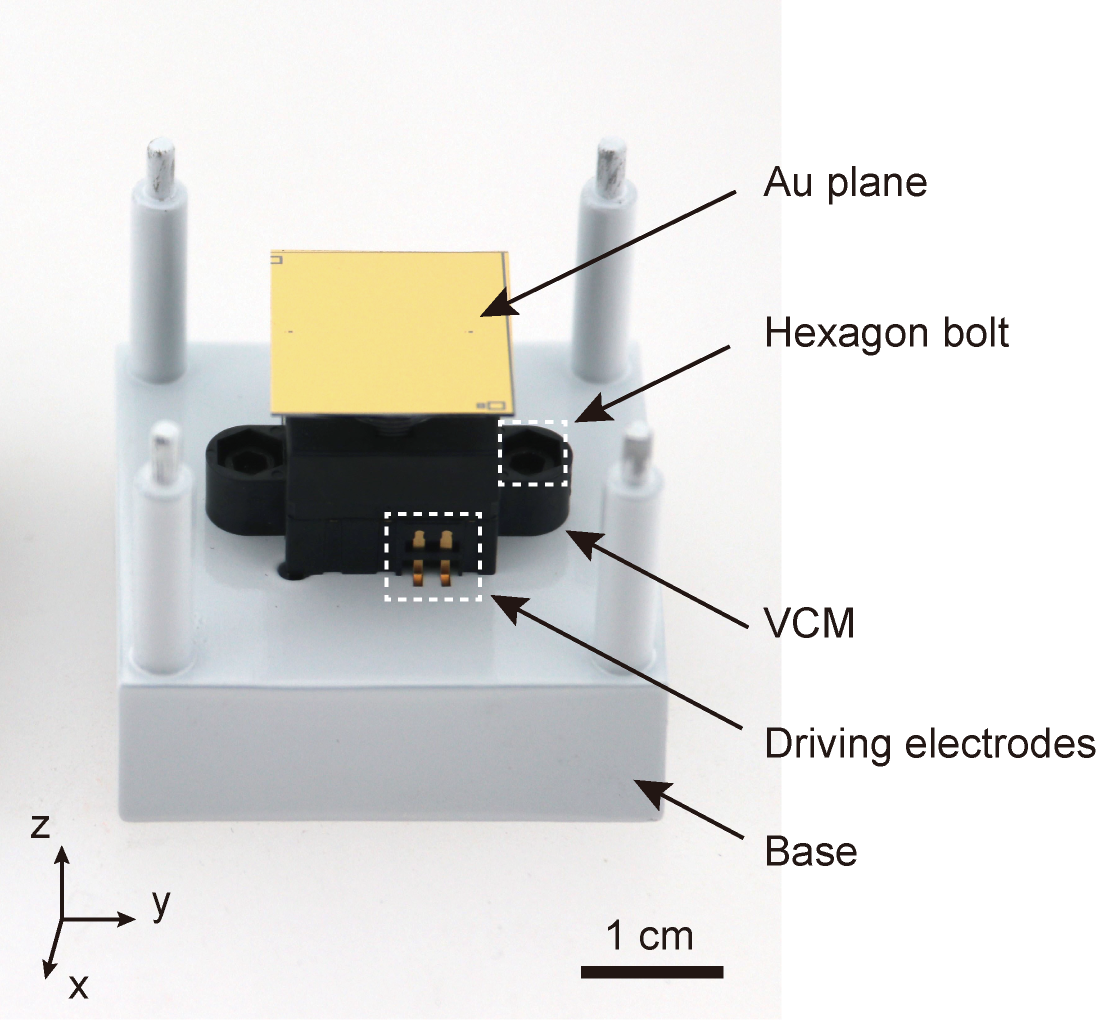


**Fig. S9** The physical drawing of the assembled MTM.

**References**

[S1] Yu N, et al. Light propagation with phase discontinuities: generalized laws of reflection and refraction. *Science* 334: 333-337 (2011).

[S2] Duan G, et al. A survey of theoretical models for terahertz electromagnetic metamaterial absorber. *Sens. Actuator A Phys.* 287, 21-28 (2019).

[S3] Sun Z, et al. WiFi energy-harvesting antenna inspired by the resonant magnetic dipole metamaterial. *Sensors* 22, 6523 (2022).

[S4] Su X, et al. Active metasurface terahertz deflector with phase discontinuities. *Opt. Express* 23, 27152-27158 (2015).

[S5] Cong L, Srivastava YK, Zhang H, Zhang X, Han J, Singh R. All-optical active THz metasurfaces for ultrafast polarization switching and dynamic beam splitting. *Light Sci. Appl.* 7, 28 (2018).

[S6] Liu M, et al. Deeply Subwavelength Metasurface Resonators for Terahertz Wavefront Manipulation. *Advanced Optical Materials* 7, 1900736 (2019).

[S7] Buchnev O, Podoliak N, Kaltenecker K, Walther M, Fedotov VA. Metasurface-Based Optical Liquid Crystal Cell as an Ultrathin Spatial Phase Modulator for THz Applications. *ACS Photonics* 7, 3199-3206 (2020).

[S8] Wu J, et al. Liquid crystal programmable metasurface for terahertz beam steering. *Applied Physics Letters* 116, 131104 (2020).

[S9] Fu X, et al. Flexible Terahertz Beam Manipulations Based on Liquid-Crystal-Integrated Programmable Metasurfaces. *ACS Appl. Mater. Interfaces* 14, 22287-22294 (2022).

[S10] Chen B, et al. Electrically addressable integrated intelligent terahertz metasurface. *Science Advances* 8, eadd1296 (2022).

[S11] Zhuang X, et al. Active terahertz beam steering based on mechanical deformation of liquid crystal elastomer metasurface. *Light Sci. Appl.* 12, 14 (2023).
